# Supplementary figures and images for: Speed-related but not detrended gait variability increases with more sensitive self-paced treadmill controllers at multiple slopes
Source: PLoS One. 2021 May 7;16(5):e0251229. doi: 10.1371/journal.pone.0251229 (PMC8104374; doi:10.1371/journal.pone.0251229)

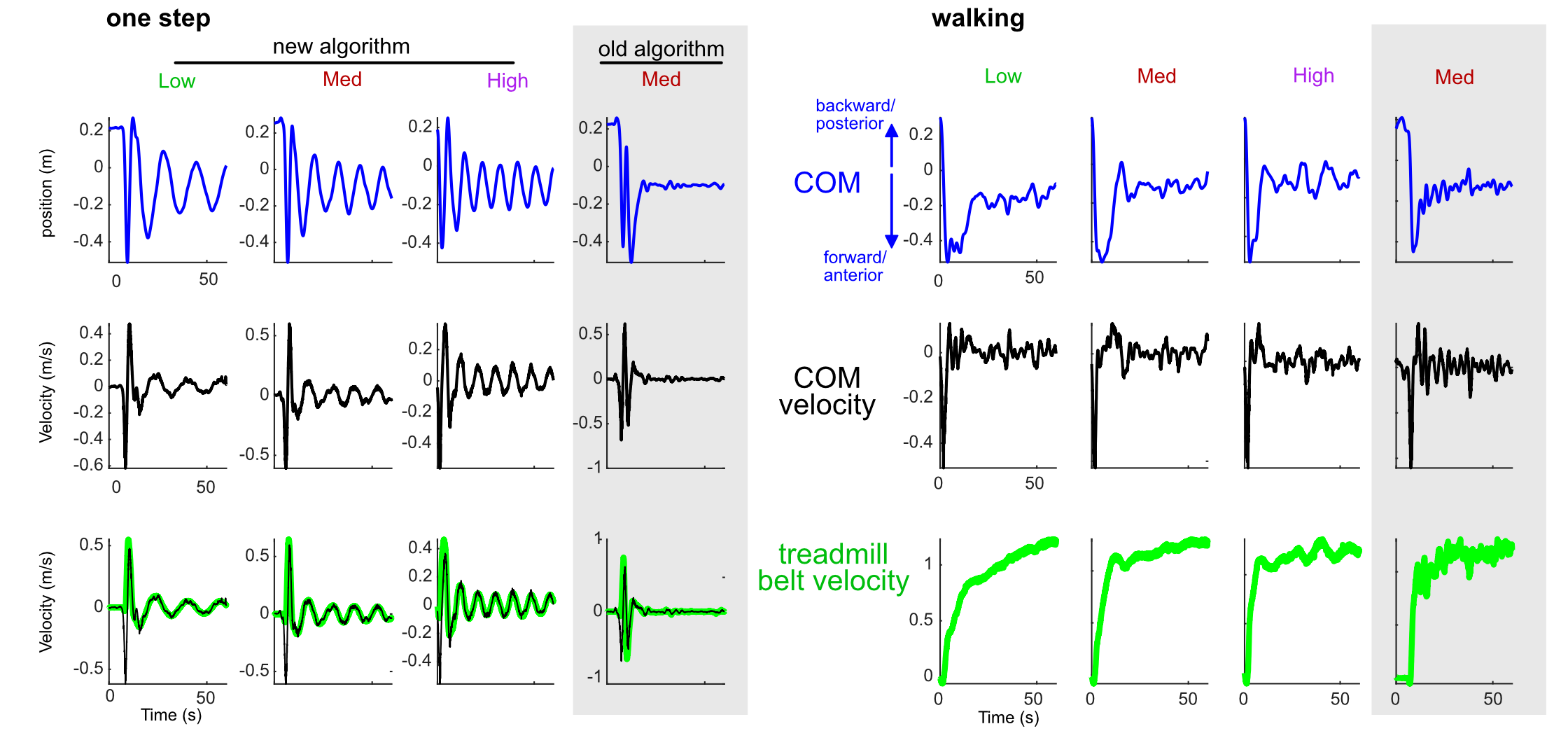

Supplement: S1 Fig — For the single step condition, the subject took a single forward step and then stood still on the treadmill belt. For the single step condition, after an initial correction in response to the change in the person’s relative position with the treadmill, the “new algorithm” produced treadmill belt velocity oscillations that resulted in COM position oscillations with a slow decay rate towards the steady state position whereas the “old algorithm” rapidly reached the steady state COM position. The COM oscillations increased in frequency for increasing sensitivities with the “new algorithm”. For the walking condition, the “old algorithm” had more frequent changes in the COM, treadmill belt velocity, and COM velocity compared to the “new algorithm”. (TIF) [file pone.0251229.s001.tif]
